# Awareness and perception of COVID-19 among the general population: A Middle Eastern survey

**DOI:** 10.1371/journal.pone.0250461

**Published:** 2021-04-22

**Authors:** Ruba M. Jaber, Baraa Mafrachi, Abdallah Al-Ani, Mustafa Shkara

**Affiliations:** 1 Department of Family and Community Medicine, School of Medicine, University of Jordan, Amman, Jordan; 2 School of Medicine, The University of Jordan, Amman, Jordan; 3 Department of Industrial Engineering, School of Applied Technical Sciences, German Jordanian University, Amman, Jordan; Chinese Academy of Medical Sciences and Peking Union Medical College, CHINA

## Abstract

Due to the sudden emergence of the novel coronavirus as a worldwide pandemic, this study aimed to evaluate the awareness and practices of both the Jordanian and Iraqi populations during the early stages of the pandemic. A cross-sectional survey was conducted between the 19^th^ and 22^nd^ of March to assess the public’s awareness toward COVID-19. Multiple scored domains were used to assess the differences between the two populations. Statistical analysis was conducted to reveal the influence of sociodemographic variables on these scores. A total of 3167 participants were recruited in the study, of which, 1599 (50.5%) were from Jordan and 1568 (49.5%) were from Iraq. More than half of the Jordanian (56.8%) and Iraqi participants (53.2%) showed average or adequate awareness about COVID-19. More than 60% of both populations relied on medical staff for COVID-19 related information. Social media was the second most common COVID-19 information source, as it was reported by 53.7% of Jordanian participants and 62.8% of Iraqi participants. More than 90% of both populations participated in precautionary measurements. Finally, about 20% of both populations failed to recognize droplet inhalation as a source of transmission. Despite the portrayed awareness levels, governmental involvement is warranted to increase the public’s awareness and fill the gaps within their knowledge.

## Introduction

A cluster of acute respiratory disease of unknown etiology emerged in Wuhan, China during late December of 2019. On the 7^th^ of January, isolation of novel coronavirus (nCoV), which was later named coronavirus disease (COVID-19) by the World Health Organization (WHO), was implicated as the cause of the outbreak. Since then, cases of COVID-19 have been on the rise over the entire globe [1].

The symptoms of the COVID-19 infection are similar to that of other beta coronaviruses [2]. Symptoms include fever, cough, dyspnea, weakness and fatigue, headache and diarrhea [3]. These symptoms appear after an incubation period, which ranges from 0 to 24 days, with a median duration of 3 days [4]. This period mainly depends on the patient’s age and immune system status. Human to human transmission is the most likely route of COVID-19 dissemination, primarily via close contact or through droplets spreading by coughing [5, 6]. The use of RNA-dependent RNA polymerase inhibitors (Remdesivir) and anti-inflammatory agents (Dexamethasone) showed promising clinical results. A recent review documented the efficacy of Remdesivir in shortening the recovery periods of infected patients. Moreover, the highest reductions of death risk and all-cause mortality were most prominently seen in patients with severe COVID-19 infection [7].

In the light of lacking consistent effective treatment, the best way to deal with the highly infective virus is by mitigating the spread of the virus. Several precautionary measurements are recommended to control the COVID-19 spread. Such recommendations include frequent hand washing, wearing a face mask, self-isolation and social distancing [4]. In early March, both Jordan and Iraq had confirmed their first cases of COVID-19, upon which the aforementioned precautionary measurement were implemented [8, 9]. This study aims to assess the awareness and perception of COVID-19 among the general populace in both Jordan and Iraq.

## Methodology

### Study setting

This study was conducted across Jordan and Iraq, which are ‘high-middle’ income countries [10, 11]. In 2018, Jordan’s population was 9.9 million, while Iraq’s population was estimated to be 38.4 million. Both countries exhibit the same male to female percentage ratio, 51.6%:49:4. The life expectancy in Jordan and Iraq is 74.3 and 70.3 years, respectively. The percentage of elderly people was 4% and 3% in Jordan and Iraq, respectively [12]. The use of internet in both Jordan and Iraq has grown dramatically during the past years. It is estimated that 87.8% and 48.3% of Jordanians and Iraqis have internet access, respectively. Facebook is the most popular social media platform in both countries, followed by Twitter [13–16].

### Study design and sampling

This cross-sectional study utilized a self-administered, online questionnaire which was distributed through social media outlets (e.g. Facebook and Twitter) across Jordan and Iraq between the 19^th^ and 22^nd^ of March. The questionnaire was created, designed and disseminated using the Google Forms platform. Participants were encouraged to fill out the form and assist in sharing the questionnaire with their family members, friends and relatives. Thus, participants were recruited by a snow ball sampling technique. Moreover, participants were limited to one response to avoid duplicated or exaggerated data. Included participants were those currently living in Jordan or Iraq, speak Arabic, had given an informed consent and successfully completed the questionnaire. Participants residing outside the aforementioned countries, and incomplete questionnaires were excluded. In the light of the current recommendations for self-isolation and home quarantine, online surveys are the most appropriate method for data collection.

### Data collection

The study tool was designed based on currently available information about COVID-19 according to the literature and available recommendations. Validity of constructs was evaluated by a professional practitioner heavily involved in public health. The questionnaire consisted of four major sections. The first section measured sociodemographic variables including gender, age, educational level and country of residence. The second section included items that are designed to measure respondents’ awareness of the following: COVID-19’s symptoms, transmission routes, precautionary measurements and possible treatment options. These aforementioned items were assessed by 27 statements; 9 for symptoms, 6 for transmission routes, 5 for precautionary measurements and 7 for possible treatment options. Participants responded to each statement with either “Yes” or “No”. A scoring system was applied, with each correct answer given one point while incorrect answers received zero points. Therefore, each item is an isolated subscale, which when combined with its complementary scales, results in a total awareness score ranging from zero to a maximum of 27 points. A pilot study was conducted to assess that reliability of the questionnaire. The Cronbach α value of all the subscales combined was approximately 0.71.

Participants were stratified into three categories based on their level of awareness: inadequate (≤18 points), average (19–20 points), and adequate (≥21 points). The validity of the chosen score categories was reached by authors’ consensus and that of experts within the field of public health. The rest of the questionnaire sections involved items that evaluates participants’ beliefs and emotions towards COVID-19. Beliefs were assessed by 3 items, while emotions were assessed by only one. Finally, participants’ self-assessment of COVID-19’s natural course and precautionary measures was assessed by 2 items.

### Data analysis

All data was exported and analyzed through the Statistical Package for Social Sciences (SPSS) version 23. A confidence interval (CI) of >95% was adopted. All of the study results are reported in descriptive statistics including means, frequencies and standard deviations (SD). Statistical differences between the various awareness subscales and gender were determined by 2-tailed Student’s *t*-test. Significant associations between various sociodemographic variables and awareness subscales were evaluated using one-way ANOVA. A p-value of <0.05 is considered statistically significant.

### Ethics approval and consent to participate

Ethical approval was obtained from the Institutional Review Board (IRB) at the University of Jordan. Participants were informed about the aim of the study, voluntary nature of participation, and their right to refuse or withdraw from the study at any point, and the potential benefit of participation in the study. All of the aforementioned information was included in a digitally written consent form that was attached with the online questionnaire. Upon accepting the conditions and statements included in the form, participants were redirected to the questionnaire. Thus, the consent form itself acted as a barrier to survey completion if rejected by the participant. This ensures that the participants whom had completed the survey, had already read and accepted its associated consent form.

## Results

### Characteristics of the sample

A total of 3167 participants had successfully filled out the online survey. Out of the studied sample, 1599 (50.5%) were from Jordan and 1568 (49.5%) were from Iraq. More than half of the Jordanian participants (56.5%) and more than third of the Iraqi participants were in the 18 to 24 years age group. Females represented the majority of participants from both Jordan and Iraq (61.1% and 71.5%, respectively). Furthermore, 70.4% of Jordanian participants and 72.4% of Iraqi participants had a bachelor or higher degrees. The sociodemographic characteristics of participants are presented in Table 1.

**Table 1:** Sociodemographic variable of 3167 participants according to country of residency and total awareness score.

Variable	Jordan					Iraq				
	Total	Total awareness score			*P value*	Total	Total awareness score			*P value*
	n (%)	Inadequate	Average	Adequate		n (%)	Inadequate	Average	Adequate	
		n (%)	n (%)	n (%)			n (%)	n (%)	n (%)	
**Gender**					0.24					0.29
Male	622(38.9)	258(41.5)	196(31.5)	168(27.0)		447(28.5)	200(44.7)	127(28.4)	120(26.9)	
Female	977(61.1)	432(44.2)	295(30.2)	250(25.6)		1121(71.5)	534(47.6)	297(26.5)	290(25.9)	
**Age group (years)**										
<18	86(5.4)	48(55.8)	23(26.7)	15(17.5)	**<0.01**	59(3.8)	30(50.8)	20(33.9)	9(15.3)	**<0.01**
18–24	903(56.5)	360(39.9)	311(34.4)	232(25.7)		573(36.5)	258(45.0)	163(28.5)	152(26.5)	
25–34	334(20.9)	139(41.6)	89(26.7)	106(31.7)		440(16.2)	191(43.4)	112(25.5)	137(31.4)	
35–44	149(9.3)	72(48.3)	37(24.8)	40(26.9)		254(16.2)	133(52.4)	63(24.8)	58(22.8)	
45–54	97(6.1)	59(60.8)	21(21.7)	17(17.5)		147(9.4)	83(56.5)	39(26.5)	25(17.0)	
55–64	23(1.4)	9(39.1)	9(39.1)	5(21.8)		75(4.8)	32(42.70	22(29.3)	21(28.0)	
>65	7(0.5)	3(42.9)	1(14.2)	3(42.9)		20(1.3)	7(35.0)	5(25.0)	8(40.0)	
**Education**					**<0.01**					**0.04**
Bachelor or higher degree	1126(70.4)	465(41.3)	353(31.4)	308(27.4)		1135(72.4)	519(45.7)	303(26.7)	313(27.6)	
High school	429(26.8)	199(46.4)	128(29.8)	102(23.8)		393(25.1)	195(49.6)	108(27.5)	90(22.9)	
Primary school	40(2.5)	23(57.5)	10(25.0)	7(17.5)		35(2.2)	16(45.7)	13(37.1)	6(17.2)	
Uneducated	4(0.3)	3(75.0)	0(0.0)	1(25.0)		5(0.3)	4(80.0)	0(0.0)	1(20.)	

### Source of awareness

**Fig 1:**
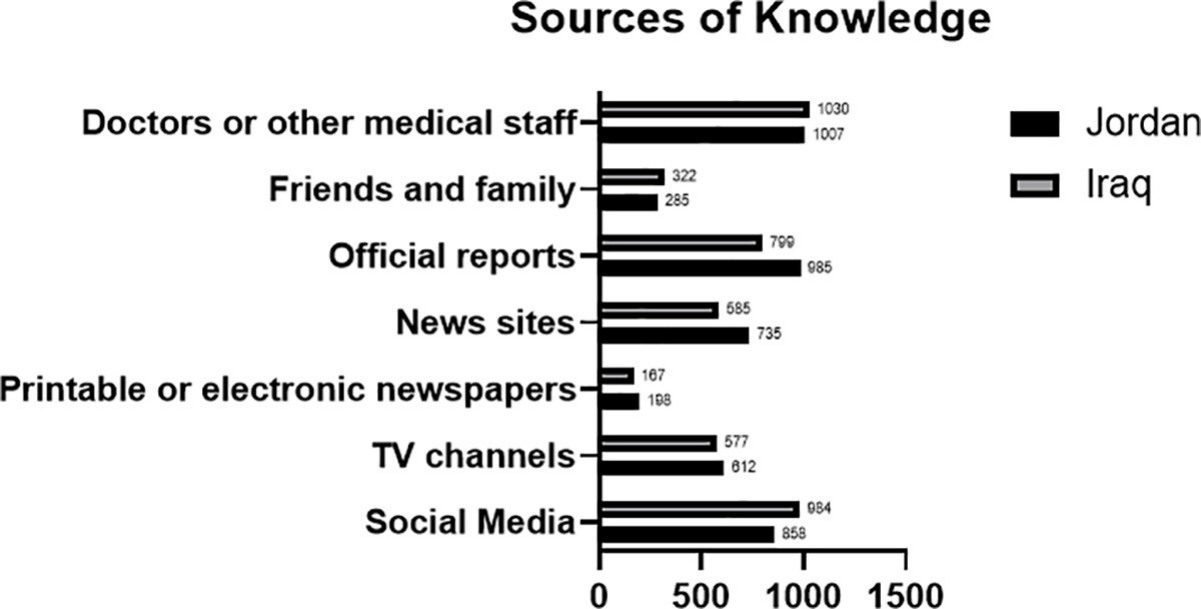
Frequency of sources of awareness use among Jordanian and Iraqi participants.

The most frequently used source of information about COVID-19 were ‘doctors and other medical staff’ among Jordanian and Iraqi participants (62.9% and 65.7%, respectively). Social media was the second most used informational medium regarding the COVID-19 pandemic as it was reported by 53.7% and 62.8% of Jordanian and Iraqi participants, respectively. On the other hand, the least frequently used information source was newspapers, which were utilized by only 12.4% and 10.7% of the Jordanian and Iraqi participants, respectively. Fig 1 illustrates the frequency of used sources of information.

### Awareness items

Table 2 demonstrates participants’ positive responses to different questionnaire items. About 55.0% of Jordanian participants and 56.0% of Iraqi participants self-reported that they are aware about the nature of COVID-19, while only 72.3% of Jordanian participants and 73.3% of Iraqi participants self-reported that they are aware about the protective and precautionary measures regarding COVID-19. Based on our results, more than half of the Jordanian participants (56.8%) and Iraqi participants (53.2%) showed an average or adequate total awareness about the novel COVID-19. Total awareness was calculated as the sum of all awareness subscales aforementioned above.

**Table 2:** Frequency of participants’ correct responses according to country of residency.

Variable	Statement	Jordan	Iraq
		n(%)	n(%)
Symptoms	Fever	1567(98.0)	1510(96.3)
	Cough	1290(80.7)	1285(82.0)
	Sneezing	537(33.6)	570(36.4)
	Dyspnea	1459(91.2)	1449(92.4)
	Fatigue and Weakness	855(53.5)	925(59.0)
	Chest pain	895(56.0)	804(51.3)
	Diarrhea	495(30.9)	348(22.2)
	Headache	1033(64.6)	918(58.5)
	Sore throat	811(50.7)	762(48.6)
Transmission route	Airborne	353(22.1)	312(19.9)
	Droplets	1339(83.7)	1291(82.3)
	Contact with contaminated surfaces	1459(91.2)	1402(89.4)
	Contaminated foods and drinks	427(26.7)	333(21.2)
	Pets	244(15.3)	150(9.6)
	Handshaking and Kissing	1491(93.6)	1427(91.0)
Precautionary measurements	Hand washing with water and soap	1480(92.6)	1405(89.6)
	Hand washing with alcohol	1213(75.9)	1109(70.7)
	Wearing face mask	948(59.3)	962(61.4)
	Avoiding crowded areas	1529(95.6)	1509(96.2)
	Avoiding handshaking and kissing	1516(94.8)	1421(90.6)
Treatments options	Currently there is no treatment	1192(74.6)	1166(74.4)
	Antibiotics	177(11.1)	223(14.2)
	Antipyretics	450(28.1)	444(28.3)
	Panadol or paracetamol	444(27.8)	458(29.2)
	Drinking hot liquids	399(24.9)	441(28.1)
	Herbal remedy	176(11.0)	61(3.9)
	Vitamins supplementation	557(34.8)	742(47.3)
Most susceptible	Pediatric	4(0.3)	5(0.3)
	Geriatric	494(30.8)	432(27.6)
	Pregnant	4(0.3)	4(0.3)
	Immunodeficient people	863(54.0)	943(60.1)
	All are susceptible equally	234(14.6)	184(11.7)
Mortality rate (%)	<5	1121(70.1)	899(57.3)
	5–20	267(16.7)	344(21.9)
	20–50	146(9.1)	209(13.3)
	50–80	54(3.4)	92(5.9)
	>80	11(0.7)	24(1.5)

Regarding the presenting symptoms of the COVID-19 infection, almost all participants from both Jordan and Iraq reported that COVID-19 presents with fever (98.0% and 96.3%, respectively). More than 90% of both Jordanian and Iraqi participants reported that dyspnea is a common symptom. About 90% of both groups believed that COVID-19 can be transmitted by handshaking and that contact with contaminated surfaces is a potential transmission route. Moreover, more than 80% of both groups of participants reported that the virus can be transmitted through droplets inhalation.

Approximately, 96% of participants from both Jordan and Iraq reported avoiding crowded areas in an attempt to minimize their risk of infection. Moreover, more than 90% of both groups reported a momentous reduction in their personal habits of handshaking and kissing. Furthermore, about 90% of participants from both Jordan and Iraq reported more frequent hand washing with soap and water. Three quarters of Jordanian and Iraqi participants reported that there is currently no cure for the novel COVID-19. On the other hand, a total of 34.8% of Jordanian participants and 47.3% of Iraqi participants believed that supplemental vitamins are potential treatment options. In addition, nearly 30% both groups believed that paracetamol is a potential treatment for COVID-19.

Approximately, 30% of both groups acknowledged that geriatric patients are the most susceptible to COVID-19 related mortality. Moreover, more than 50% of both groups believed that the mortality rate associated with COVID-19 is less than 5%.

### Sociodemographic variables and awareness subscales

Our results showed a statistically significant difference in precautionary measurements mean score among the two included genders in the Iraqi cohort (p<0.01). Females were more likely to have higher means of precautionary measurement scores as compared with their male counterparts (Mean Difference: 0.1903; Confidence Interval: 0.07–0.3). Moreover, statistically significant associations were demonstrated among participants from both Jordan and Iraq between different age groups and transmission, precautionary measurement, and treatment scores (all p≤ 0.05). Post-hoc analysis failed to produce consistent trends among different ages groups with the various awareness subscales. In addition, the results showed a statistically significant association between educational level and treatment score among Jordanian participants (p<0.01). In which, participants with higher educational levels were found to have higher awareness scores with regards to the treatment score subscale. On the other hand, educational level and symptoms score were statistically significant among Iraqi participants (p = 0.02). Similar associations between transmission scores among Iraqis and precautionary scores among Jordanians with higher educational levels were also demonstrated (p<0.01, p<0.01), respectively. Table 3 presents the study’s sociodemographic variables and their association with different awareness subscales.

**Table 3:** Statistical associations between different sociodemographic variables and awareness subscales.

Awareness subscales	Jordan		Iraq	
	Education*	Age groups*	Education*	Age groups*
Symptoms Score	0.47	0.12	**0.02**	0.28
Transmission Score	0.20	**0.04**	**<0.01**	**<0.01**
Precautionary Score	**<0.01**	**<0.01**	0.09	**<0.01**
Treatment Score	**<0.01**	**<0.01**	0.07	**<0.01**

The values in each cell corresponds to the p-value of the possible association examined by the study.

*Associations of the following variables and the various awareness subscales were examined by one-way ANOVA.

## Discussion

The emerging COVID-19 pandemic is a distinct period that is infested with a variety of social, economic and health challenges. The affected populations need to have an adequate level of COVID-19 related awareness, in order to meet the aforementioned challenges and form realistic expectations regarding the disease’s future course. The health-related and socioeconomic burden of the pandemic might prove to be disastrous, especially it is to be mishandled by the public and disregarded by concerned authorities. Since the entirety of any population that is contact with COVID is vulnerable to its consequences, this study was conducted to evaluate the awareness, perceptions and attitudes of the general public in Jordan and Iraq towards the early spread of COVID-19.

The results of our survey showed that both medical staff and social media platforms are the main sources of information, from which participants seek COVID-19 related knowledge. This highlights the vital role of health care workers in providing accurate and reliable information regarding the virus. Not surprisingly, social media is an important source of information due to its easy accessibility and widespread. However, given the novelty of COVID-19 and its rapid spread worldwide, the internet and social media platforms are supersaturated with information about the virus, yet a huge portion of this information is misleading [17]. It’s evident from the literature that both social media and health care workers are the primary sources of information during times of crisis [18–21]. However, studies show that European respondents from Netherlands, Germany and Italy, and Australian citizens utilized traditional media (e.g., television, and news) as their primary sources of information regarding the COVID-19 pandemic [22, 23]. Such phenomenon could be attributed to the fact that in these countries, official governmental reports regarding the virus are broadcasted on traditional media.

Participants’ self-reported assessment nearly reflected their actual knowledge and awareness, which is evident from the congruence between self-assessment and awareness score percentages, which are supported by statistical analysis (p<0.01). In general, differences in awareness and practices between Jordanian and Iraqi participants were minimal. Participants from both countries were fairly aware of the COVID-19 symptoms, which closely resemble that of flu illnesses. However, only 80% of the participants were aware that COVID-19 can be transmitted by droplets, despite the fact that droplet transmission is already established as its main transmission route [5]. This high percentage of unaware participants could be attributed to misleading information on social media platforms [24]. Moreover, participants, who are unaware of the transmission route, are more likely to underestimate the importance of the recommended preventative measurements which include face masks usage, social distancing, and commitment to proper hygiene practices, resulting in less adherence to such vital measures and increased dissemination of the virus. This finding points out the importance of providing accurate and trustworthy information about the novel virus by health care authorities, health care workers and other involved sectors through the use of both official platforms and social media. However, even with the presence of some recommended therapies such as Remdesivir and Dexamethasone, the promotion and implementation of preventative measurements is still the single most important and widely recommended intervention to mitigate the virus’s propagation.

The general populace of both countries demonstrated significant commitment to the implementation of the recommended preventive measurements; however, these high rates are possibly influenced by the strict governmental orders. These findings are comparable to the results of recent studies conducted in several western nation including USA and Europe, in which the studies’ respondents demonstrated high rates of compliance with the implementation of preventative practices and measurements [22, 23, 25]. Although, Jordanian and Iraqi participants exhibited an acceptable level of general awareness, they held vague preconceptions about the possible treatment options. Only two-thirds of the participants are aware that there is no cure or vaccine at the time in which this study was conducted. Moreover, a substantial proportion showed a false belief regarding the effectiveness of vitamin supplements and herbal remedies as a treatment for COVID-19, which is a widespread tradition in the middle east region. These findings are consistent with a study conducted in Saudi Arabia during the MERS outbreak [26].

Our study shows that there is a significant difference between genders with respect to the examined precautionary scores. Post-hoc analysis portrays that females have higher scores in terms of awareness and compliance towards precautionary measurements. This finding supported by a recent study conducted throughout several European countries and Middle Eastern countries such as Jordan, Iraq, and Palestine [8, 9, 23, 27]. Females tend to display more protective behaviors even in hypothetical conditions similar to that of a pandemic, which was previously documented by a recent Jordanian report on the predictors of health preventive measures [28, 29]. In addition, participants with higher degrees of education seem to have a better understanding of COVID treatments as portrayed by their higher scores. Such observation seems appropriate as participants with higher degrees are more likely to self-educate themselves. Similar results were demonstrated in the literature among different Middle Eastern countries and China [8, 9, 19, 24, 27, 30, 31]. Finally, younger age seems to statistically correlate with higher treatment, precautionary and transmission scores. However, this observation might be an inherit limitation of that data collection tool as online surveys are more popular towards a younger pool of participants.

The study’s methodology, despite being appropriate for response collection during an active pandemic, may fall prey to a number of inherit biases. Online surveys are usually engaging to younger populations which might excluded or underestimated the responses of older participants due to their limited accessibility to technology. Moreover, the use of online surveys, that are primarily disseminated through social media platforms, enabled us to recruit a larger and more diverse sample of participants within a short period of time [32]. However, they may have resulted in a demographic selection bias, manifesting as an over representation of females and younger age groups, while under representing older people and those with limited internet or social media access [33]. The close ended nature of the study’s questionnaire might have missed significant unavailable responses or failed to accommodate for the wide ranges of perspectives and attitudes associated with pandemics. Finally, the cross-sectional design of the study might hinder the robustness of the associations observed.

## Conclusion

In summary, the degree of awareness portrayed by the studied populations is mildly adequate, yet lacking in specific dimensions such as transmission and treatment awareness. It seems that both populations exhibit similar trends of attitudes and perceptions toward the COVID-19 pandemic, hence their analogues cultures. Concerned authorities should allocate time and resources into raising awareness through official platforms and extensive social media campaigns, in an effort to help the populace overcome the challenges of the emergent pandemic.

## Supporting information

S1 Survey Awareness and perception of COVID-19 questionnaire. (DOCX)

S1 Data Data from Jordanian respondents. (XLSX)

S2 Data Data from Iraqi respondents. (XLSX)
