# Supplementary material for: Awareness and perception of COVID-19 among the general population: A Middle Eastern survey
Source: PLoS One. 2021 Apr 22;16(4):e0250461. doi: 10.1371/journal.pone.0250461 (PMC8062075; doi:10.1371/journal.pone.0250461)
Supplement: S1 Survey — (DOCX) [file pone.0250461.s001.docx]

**The original survey**

1. **In English**

**Gender:**

- Male
- Female

**Age:**

- Less than 18
- 18-24 year
- 25-34 year
- 35-44 year
- 45-54 year
- 55-64 year
- 65 years or greater

**Country of residency:**

- Jordan
- Iraq

**Educational level:**

- Uneducated
- Primary school
- Secondary school
- Bachelor’s or higher degree

**Do you think you know enough about COVID-19 nature?**

- No
- Yes
- Maybe

**Do you think you know enough about COVID-19 Precautionary measurements?**

- No
- Yes
- Maybe

**What information’s sources do you use to get knowledge about COVID-19?**

- Social media
- Television
- News sites
- Electronic and printable newspaper
- Official reports
- Family and friends
- Doctors and medical staff

**What are the COVID-19 infection symptoms?**

- Fever
- Cough
- Sneezing
- Dyspnea
- Fatigue and weakness
- Chest pain
- Diarrhea
- Headache
- Sore throat

**What is the most serious symptom of COVID-19 infection?**

- Fever
- Cough
- Sneezing
- Dyspnea
- Fatigue and weakness
- Chest pain
- Diarrhea
- Headache
- Sore throat

**What is the possible transmission route of COVID-19?**

- Airborne
- Droplets
- Contact with contaminated surfaces
- Contaminated foods and drinks
- Pets
- Handshaking and kissing

**What are the precautionary measurements to reduce the risk of COVID-19 infection?**

- Hand washing with water and soup
- Hand washing with alcoholic disinfectant
- Face masks
- Avoiding crowded area
- Avoiding handshaking and kissing

**What are the possible treatment options for COVID-19 infection?**

- There is currently no treatment
- Antibiotics
- Antipyretics
- Panadol and paracetamol
- Drinking hot drinks
- Herbal remedy
- Vitamins

**What are the consequences of COVID-19 infection?**

- May lead to death
- Organ failure
- Immunodeficiency
- Permanent disability
- There is no side effect

**What is the mortality rate of COVID-19?**

- 1%
- 1-5%
- 5-20%
- 20-35%
- 35-50%
- 50-65%
- 65-80%
- 80-100%

**Who is the most susceptible group to death due to COVID-19?**

- Pediatric
- Geriatric
- Pregnant women
- Immunodeficient people
- All are susceptible

**How COVID-19 outbreak did affect your emotional status?**

- Sad
- Happy
- Zealot
- Depressed
- Worried
- Panic
- Didn’t affect me

1. **In Arabic**
2. **الجنس** :

- ذكر
- انثى

1. **العمر** :

- اقل من 18 سنة
- 18-24 سنة
- 25-34 سنة
- 35-44 سنة
- 45-54 سنة
- 55-64 سنة
- 65 سنة او اكبر

1. **مكان السكن:**

- الاردن
- العراق

1. **التحصيل العلمي:**

- غير متعلم
- الابتدائية
- الثانوية العامة (البكلوريا)
- شهادة جامعية او اعلى

1. **هل تعتقد بانك على علم كافي بطبيعه فايروس الكورونا المستجد ؟**

- نعم
- لا
- ربما

1. **هل تعتقد بانك على علم كافي بطرق الوقايه من فايروس كورونا المستجد ؟**

- نعم
- لا
- ربما

1. **ما هي المصادر التي تاخذ منها معلوماتك عن الفايروس المستجد:**

- وسائل التواصل الاجتماعي
- محطات التلفاز
- المواقع الاخبارية
- الجرائد الورقية او الالكترونية
- التقارير الرسمية الصادرة عن الدولة
- الاهل والاصدقاء
- الكوادر الطبية

1. **بأعتقادك الشخصي, ماهي اعراض فايروس الكورونا المستجد؟**

- ارتفاع درجه الحرارة
- السعال
- العطاس
- صعوبه في التنفس
- تعب و خمول
- الم في الصدر
- أسهال
- صداع
- التهاب الحلق

1. **بأعتقادك الشخصي, ما هو اخطر عرض لفايروس كورونا ؟**

- ارتفاع درجه الحرارة
- السعال
- العطاس
- صعوبه في التنفس
- تعب و خمول
- الم في الصدر
- أسهال
- صداع
- التهاب الحلق

1. **بأعتقادك الشخصي, ماهي طرق انتقال فايروس كورونا ؟**

- عن طريق الهواء المستنشق
- عن طريق الرذاذ المتطاير
- عن طريق ملامسه الاسطح المولوثه
- عن طريق الاكل او الشرب
- بواسطه الحيوانات المنزليه
- عن طريق مصافحه او ملامسه شخص

1. **بأعتقادك الشخصي, ماهي طرق الوقايه المناسبه لتجنب الاصابه بفايروس كورونا ؟**

- غسل اليدين بالماء و الصابون
- غسل اليدين بالمطهرات الكحوليه
- ارتداء الكمامات
- الابتعاد عن الاماكن المكتضه بالسكان
- التخفيف من المصافحه باليدين او التقبيل

1. **بأعتقادك الشخصي, ماهي طرق علاج فايروس كورونا المستجد ؟**

- لا يوجد علاج لحد الان
- مضادات حيويه
- خافض حراره
- بنادول او براسيتامول
- شرب السوائل الحارة
- العلاج بالاعشاب
- تناول الفيتامينات

1. **بأعتقادك الشخصي, ماهي الاثار الجانبيه للاصابه بفايروس كورونا ؟**

- قد يؤدي الى الوفاة
- فشل في اعضاء الجسم
- نقص في المناعه
- الاعاقه الدائمه
- لايوجد اثار جانبيه

1. **بأعتقادك الشخصي, ماهي نسبه الوفاه للاشخاص المصابين بفايروس كورونا ؟**

- 1%
- 1-5%
- 5-20%
- 20-35%
- 35-50%
- 50-65%
- 65-80%
- 80-100%

1. **بأعتقادك الشخصي, من هم الاشخاص الاكثر عرضه للوفاة نتيجه الاصابه بفايروس كورونا؟**

- الاطفال
- كبار السن
- النساء الحوامل
- الاشخاص قليلي المناعه
- الجميع معرض للوفاة

1. **كيف اثر انتشار فايروس كورونا المستجد على صحتك النفسيه ؟**

- حزين
- سعيد
- متعصب
- مكتئب
- قلق
- في حاله ذعر
- لم يؤثر علي

1. **The survey as seen by respondents**


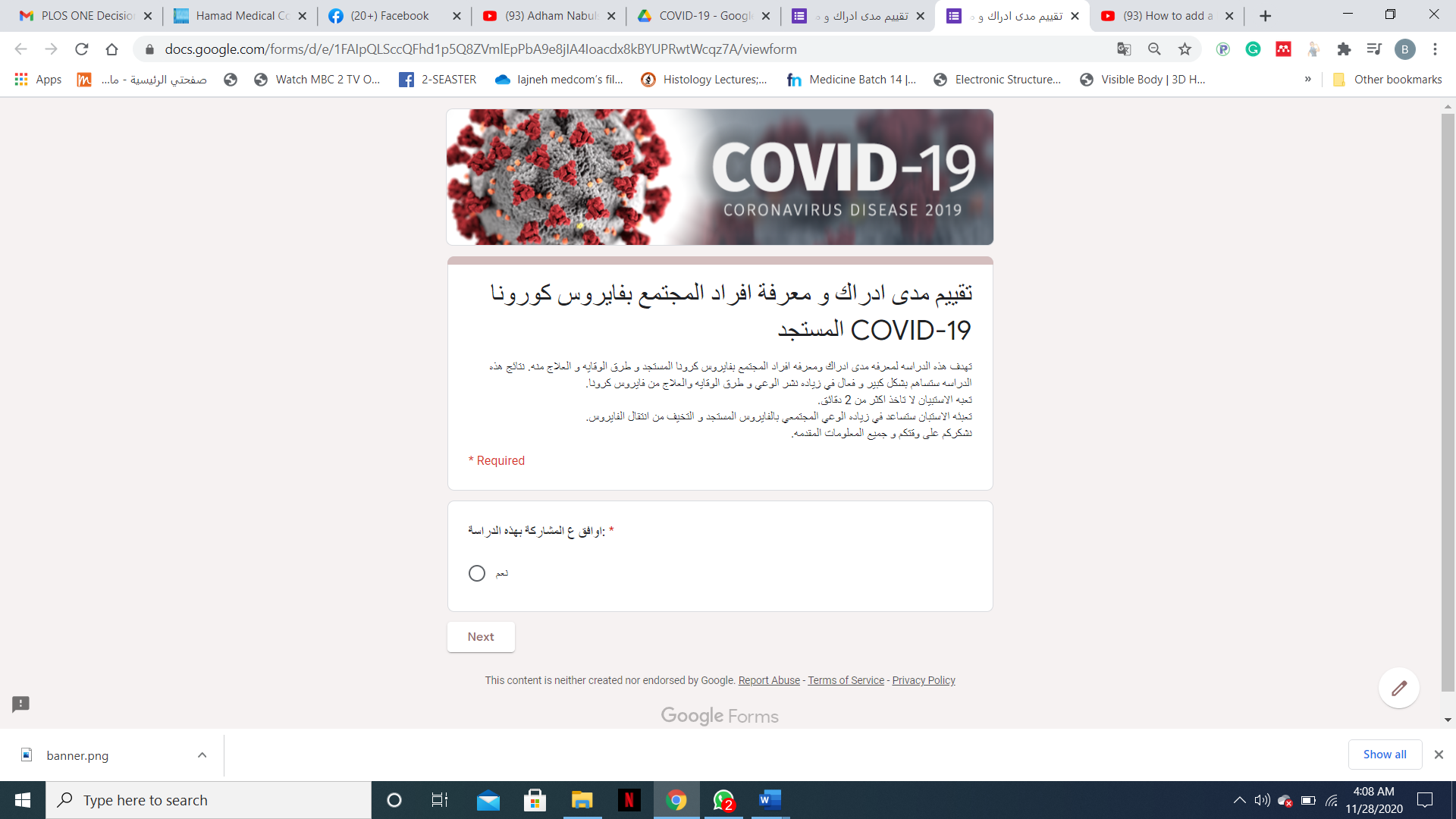


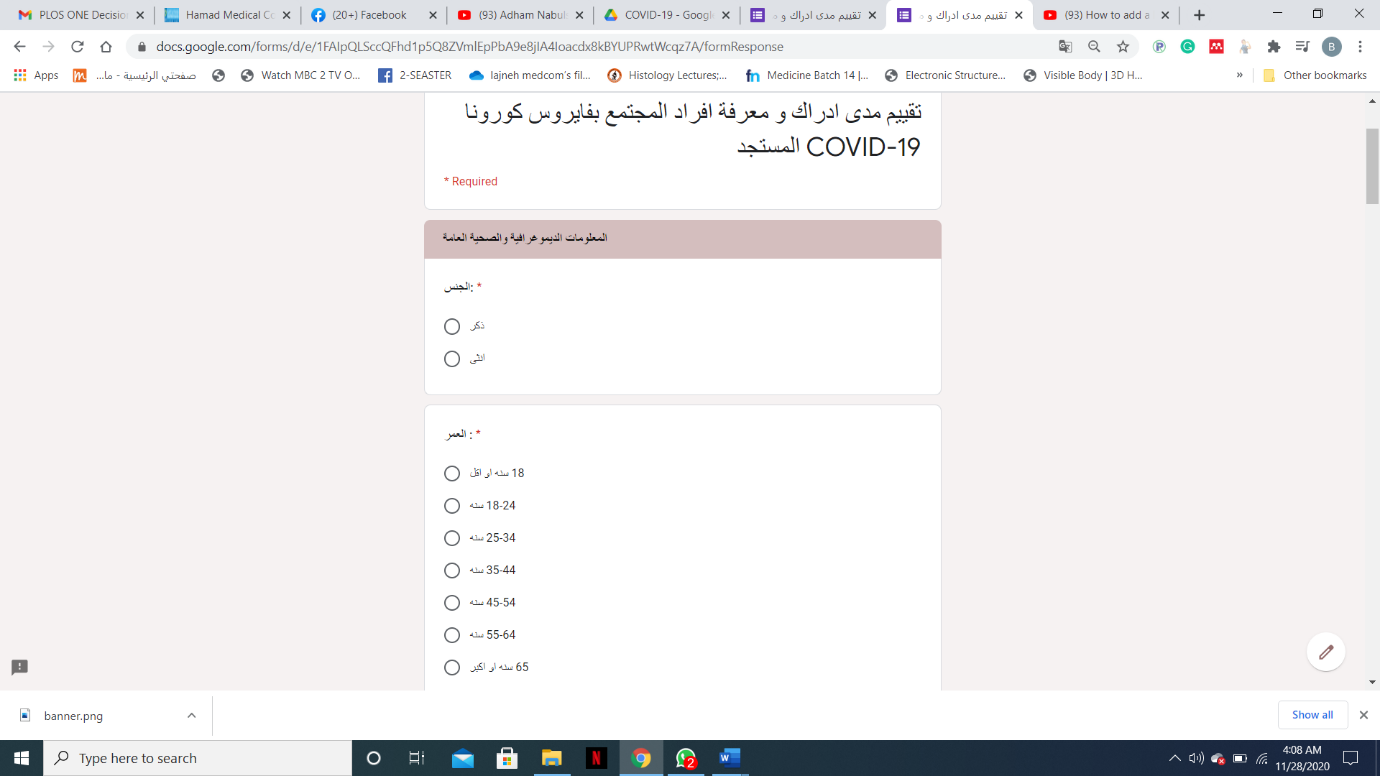


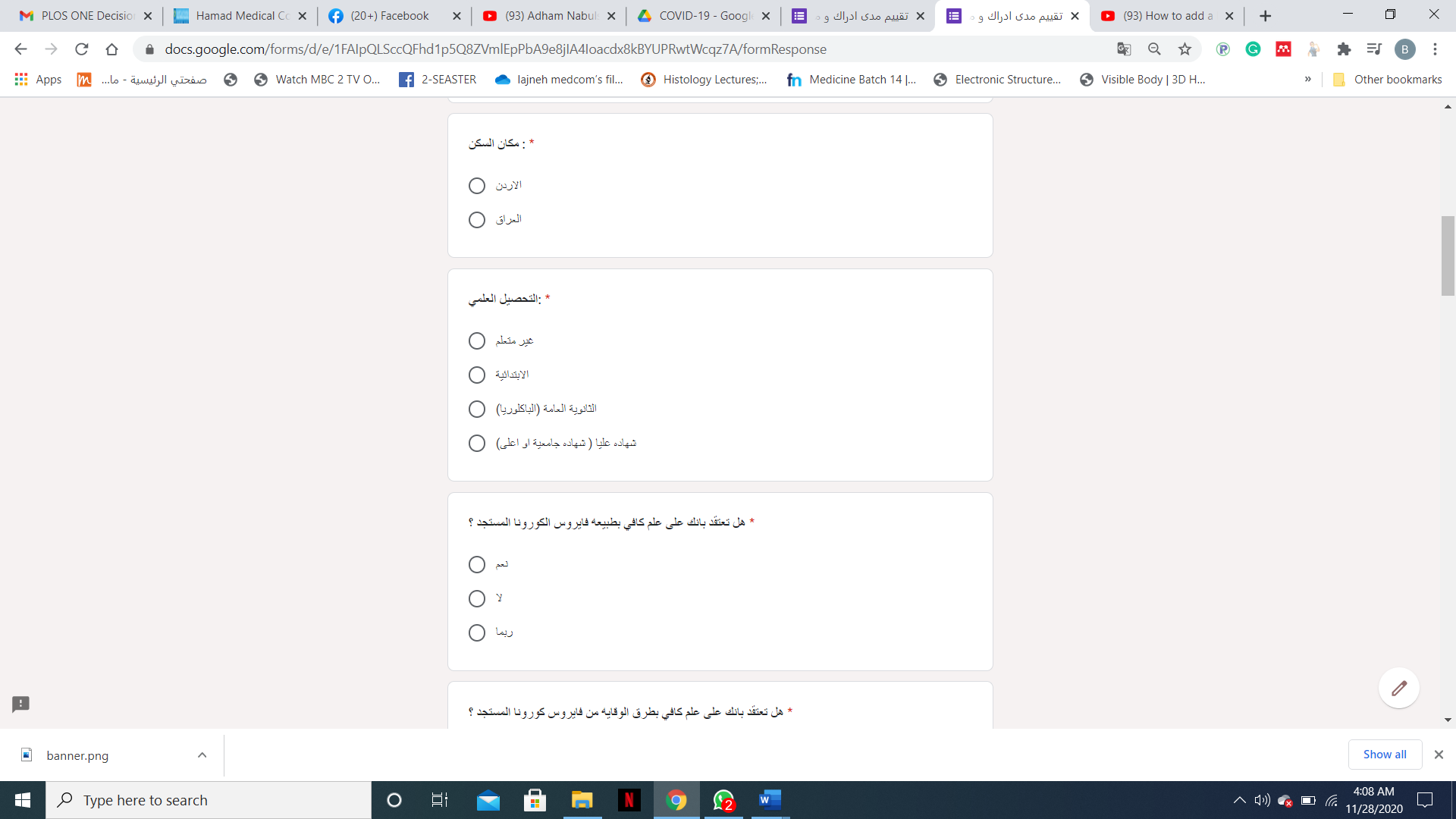


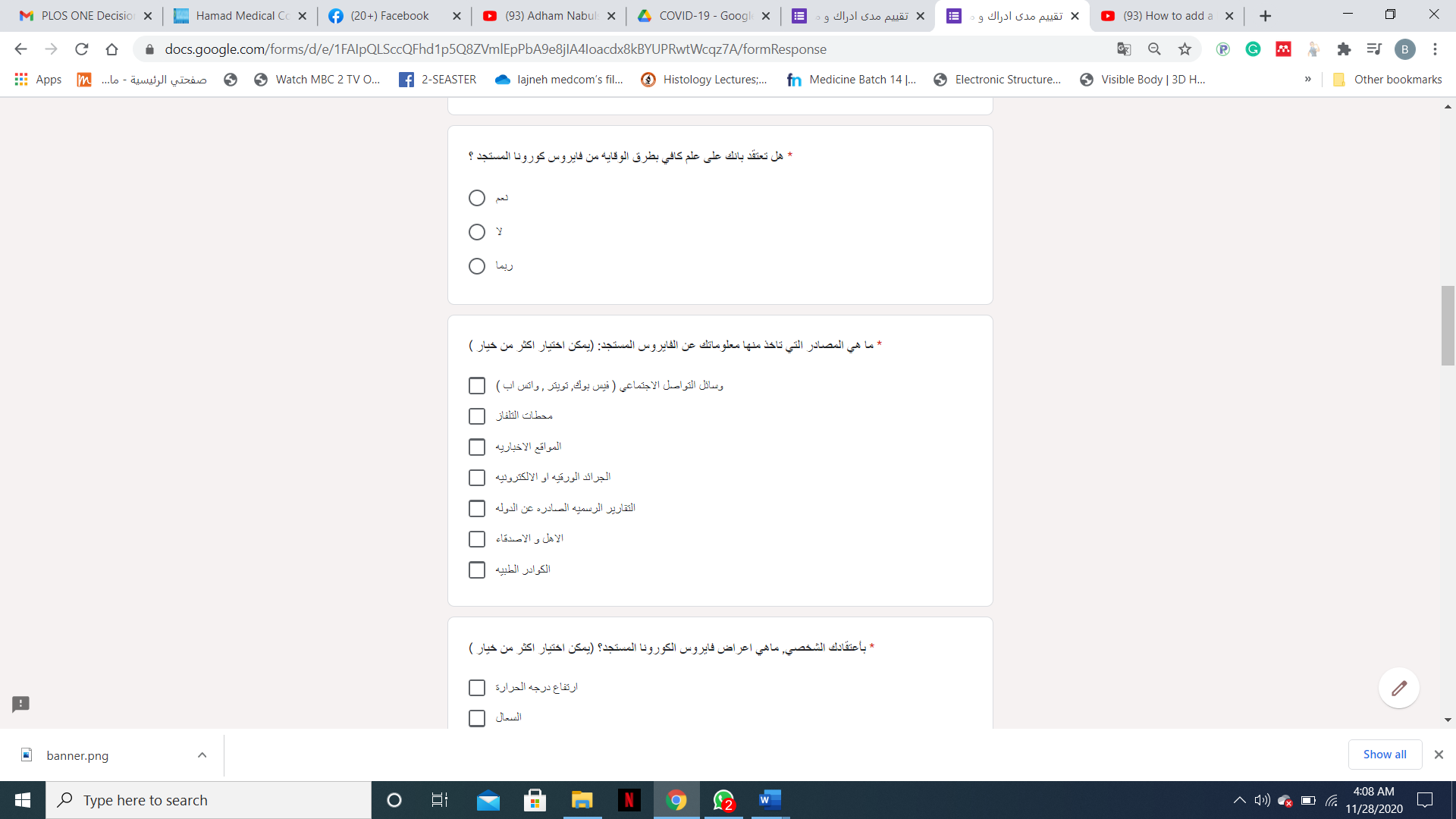


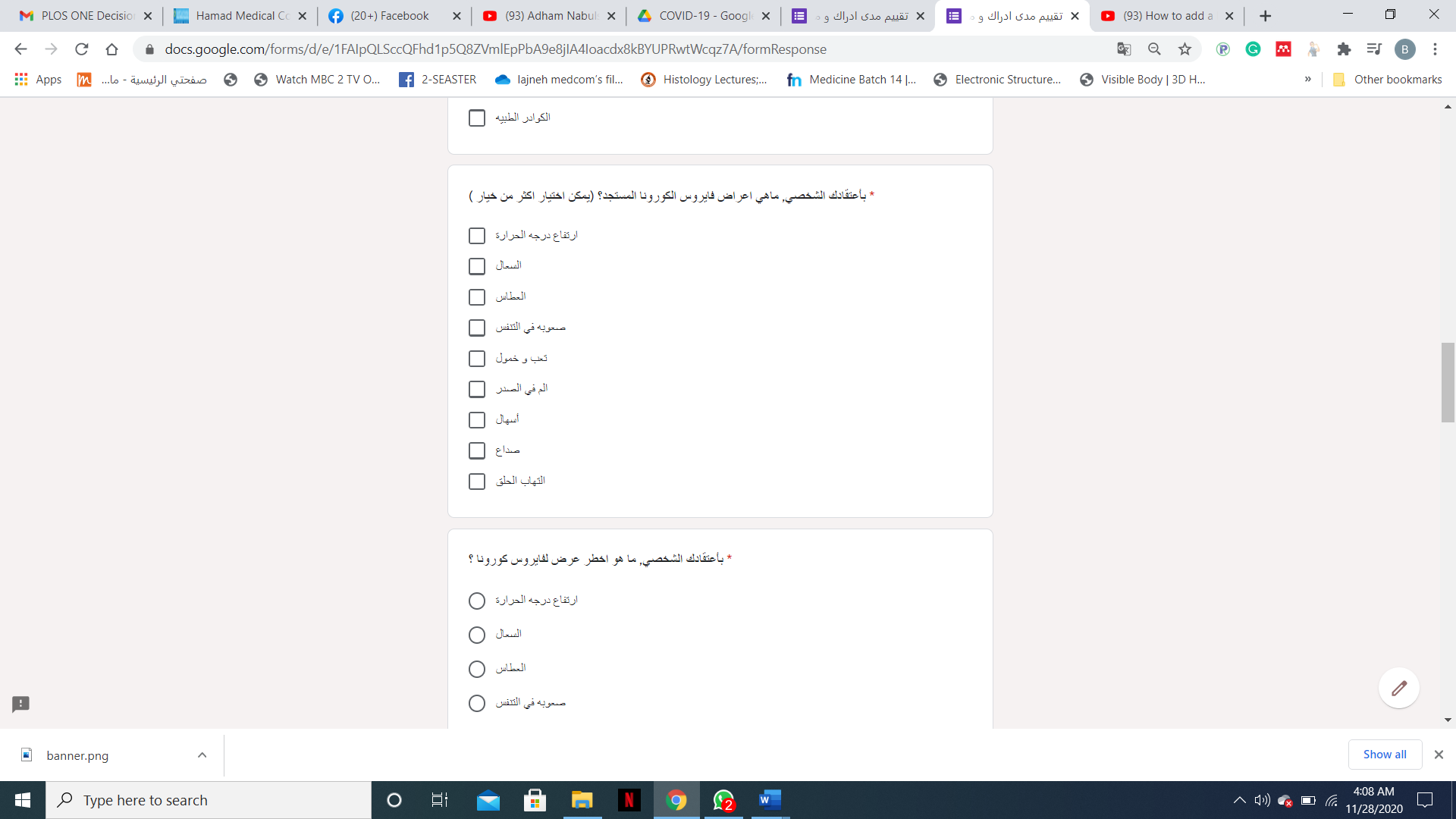


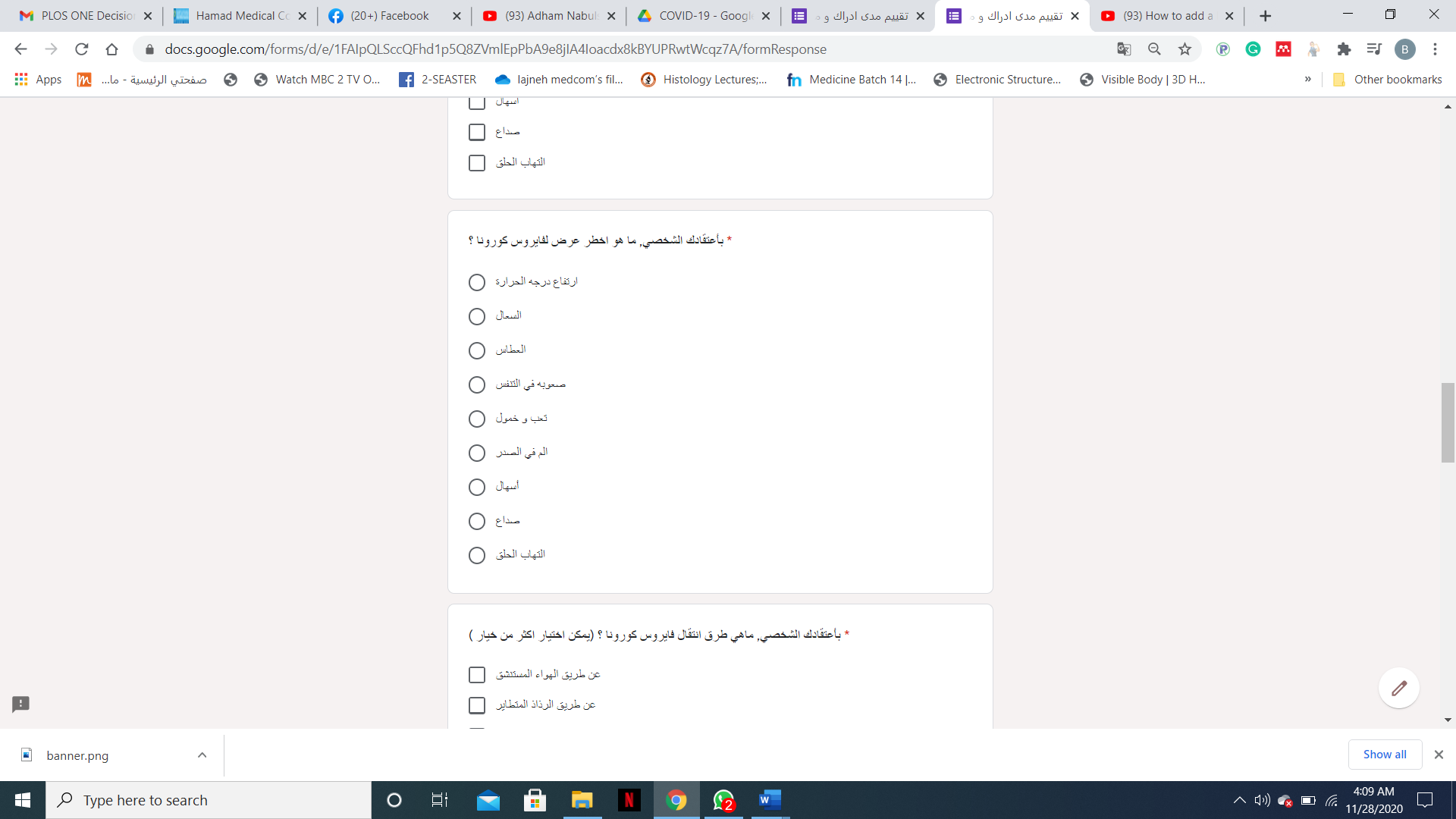


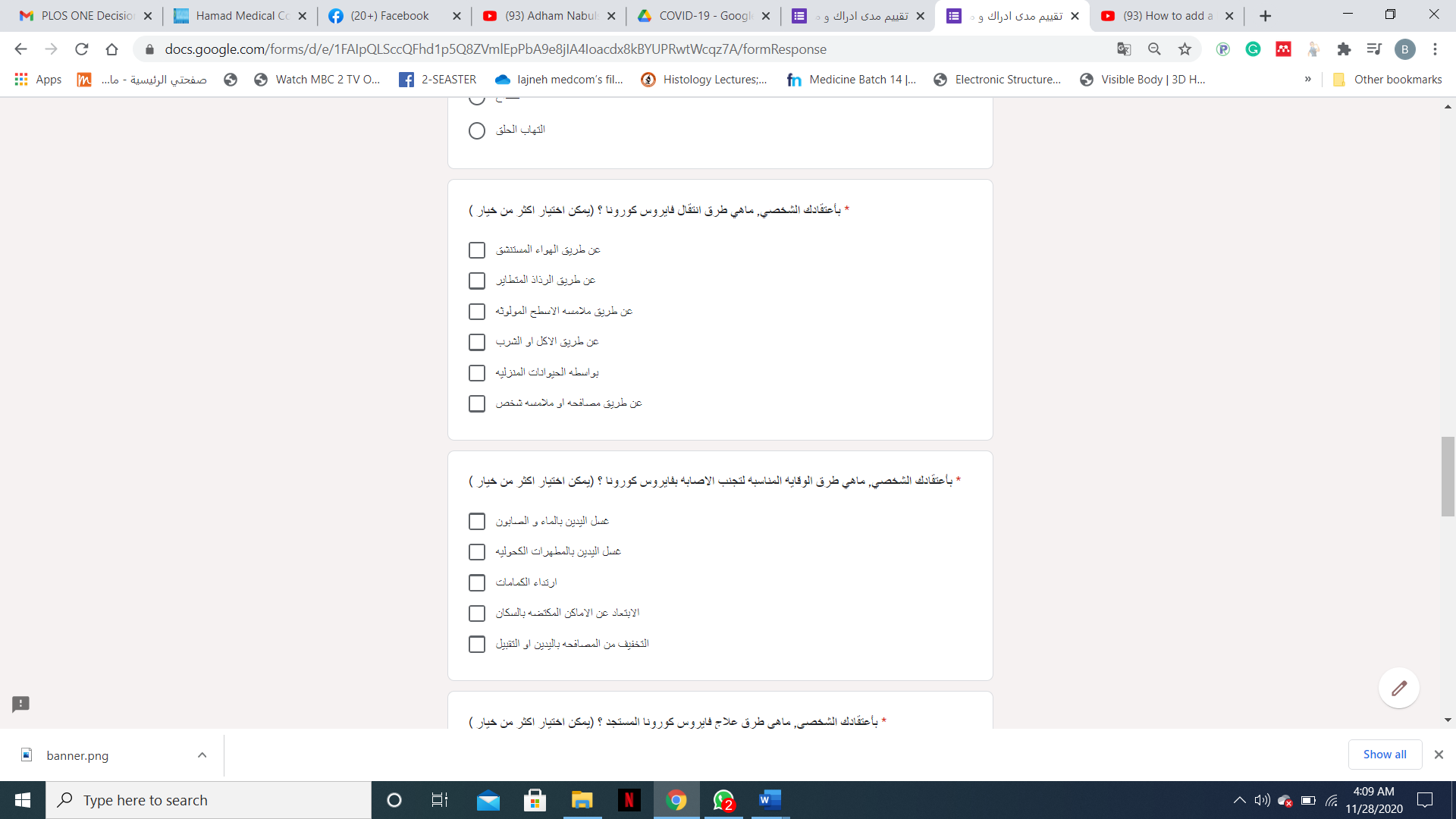


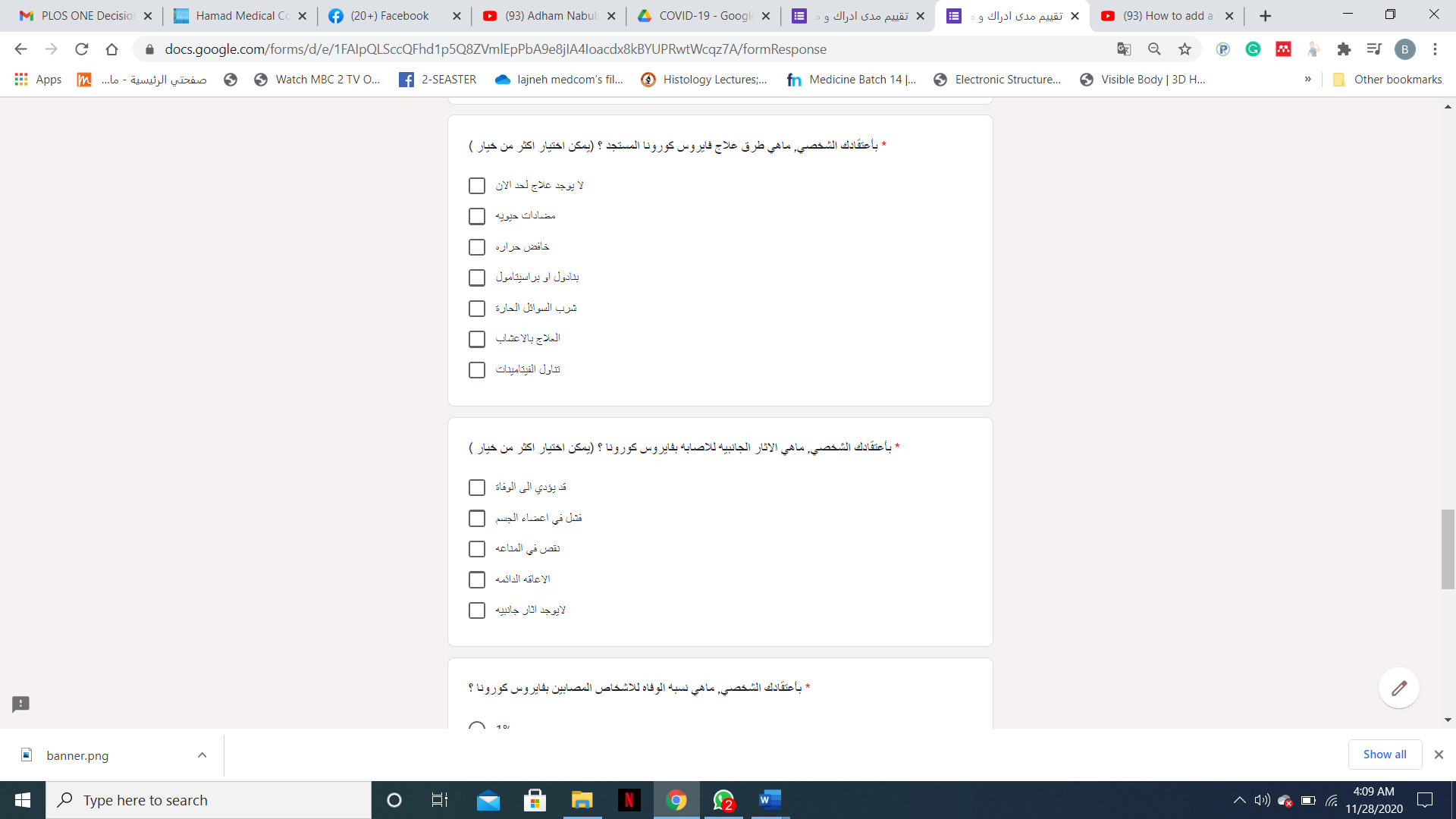


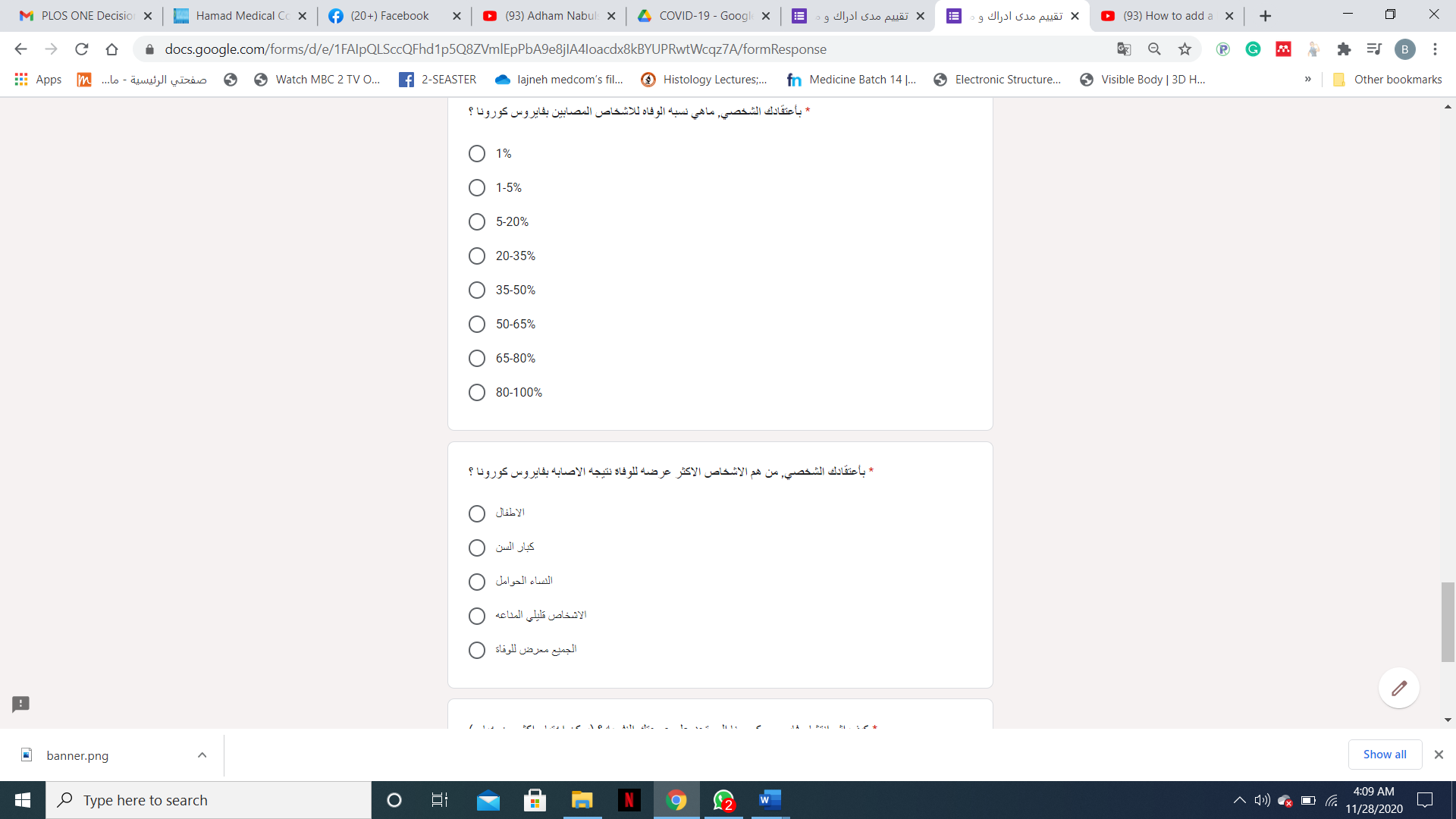


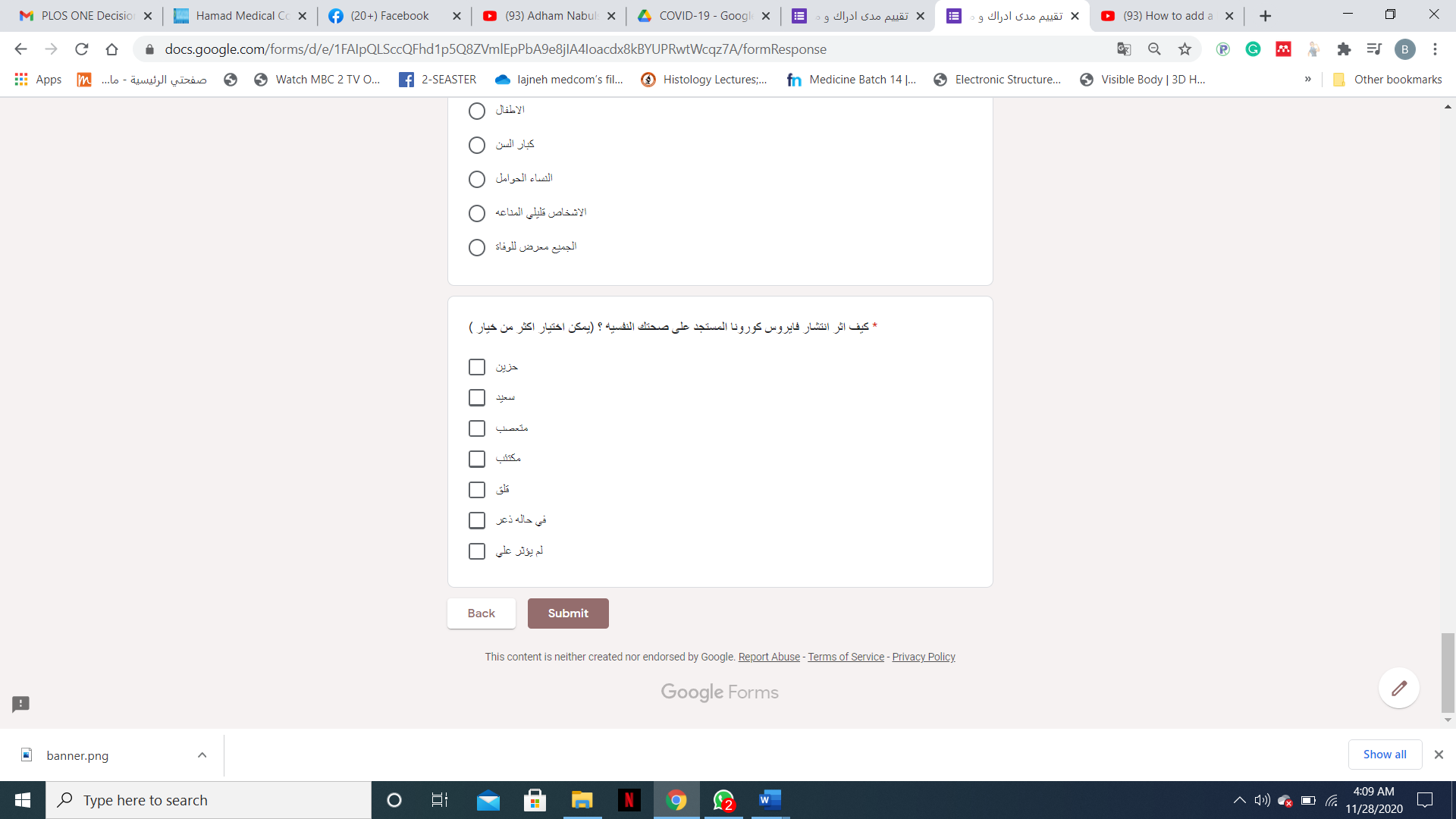


1. **Data coding and scoring**

**Gender**

- Male (0)
- Female (1)

**Age:**

- Less than 18 (0)
- 18-24 year (1)
- 25-34 year (2)
- 35-44 year (3)
- 45-54 year (4)
- 55-64 year (5)
- 65 years or greater (6)

**Country of residency:**

- Jordan (0)
- Iraq (1)

**Educational level:**

- Uneducated (0)
- Primary school (1)
- Secondary school (2)
- Bachelor’s or higher degree (3)

**Do you think you know enough about COVID-19 nature?**

- No (0)
- Yes (1)
- Maybe (2)

**Do you think you know enough about COVID-19 Precautionary measurements?**

- No (0)
- Yes (1)
- Maybe (2)

**What information’s sources do you use to get knowledge about COVID-19?**

- Social media (No 0, Yes 1)
- Television (No 0, Yes 1)
- News sites (No 0, Yes 1)
- Electronic and printable newspaper (No 0, Yes 1)
- Official reports (No 0, Yes 1)
- Family and friends (No 0, Yes 1)
- Doctors and medical staff (No 0, Yes 1)

**What is the COVID-19 infection symptoms?**

- Fever (0)
- Cough (1)
- Sneezing (2)
- Dyspnea (3)
- Fatigue and weakness (4)
- Chest pain (5)
- Diarrhea (6)
- Headache (7)
- Sore throat (8)

**What is the most serious symptom of COVID-19 infection?**

- Fever (0)
- Cough (1)
- Sneezing (2)
- Dyspnea (3)
- Fatigue and weakness (4)
- Chest pain (5)
- Diarrhea (6)
- Headache (7)
- Sore throat (8)

**What is the possible transmission route of COVID-19?**

- Airborne (No 0, Yes 1)
- Droplets (No 0, Yes 1)
- Contact with contaminated surfaces (No 0, Yes 1)
- Contaminated foods and drinks (No 1, Yes 0)
- Pets (No 1, Yes 0)
- Handshaking and kissing (No 0, Yes 1)

**What are the precautionary measurements to reduce the risk of COVID-19 infection?**

- Hand washing with water and soup (No 0, Yes 1)
- Hand washing with alcoholic disinfectant (No 0, Yes 1)
- Face masks (No 0, Yes 1)
- Avoiding crowded area (No 0, Yes 1)
- Avoiding handshaking and kissing (No 0, Yes 1)

**What are the possible treatment options for COVID-19 infection?**

- There is currently no treatment (No 0, Yes 1)
- Antibiotics (No 1, Yes 0)
- Antipyretics (No 0, Yes 1)
- Panadol and paracetamol (No 0, Yes 1)
- Drinking hot drinks (No 1, Yes 0)
- Herbal remedy (No 1, Yes 0)
- Vitamins (No 1, Yes 0)

**What are the consequences of COVID-19 infection?**

- May lead to death (0)
- Organ failure (1)
- Immunodeficiency (2)
- Permanent disability (3)
- There is no side effect (4)

**What is the mortality rate of COVID-19?**

- 1% (0)
- 1-5% (1)
- 5-20% (2)
- 20-35% (3)
- 35-50% (4)
- 50-65% (5)
- 65-80% (6)
- 80-100% (7)

**Who is the most susceptible group to death due to COVID-19?**

- Paediatric (0)
- Geriatric (1)
- Pregnant women (2)
- Immunodeficient people (3)
- All are susceptible (4)

**How COVID-19 outbreak did affect your emotional status?**

- Sad (0)
- Happy (1)
- Zealot (2)
- Depressed (3)
- Worried (4)
- Panic (5)
- Didn’t affect me (6)

**Symptoms SCORE: (out of 9 points):**

| **Measure** | **Yes** | **No** |
| --- | --- | --- |
| Fever | 1 | 0 |
| Cough | 1 | 0 |
| Sneezing | 1 | 0 |
| Dyspnea | 1 | 0 |
| Fatigue and weakness | 1 | 0 |
| Chest pain | 1 | 0 |
| Diarrhea | 1 | 0 |
| Headache | 1 | 0 |
| Sore throat | 1 | 0 |

**Transmission SCORE: (out of 6 points):**

| **Measure** | **Yes** | **No** |
| --- | --- | --- |
| Airborne | 1 | 0 |
| Droplet | 1 | 0 |
| Contaminated surface | 1 | 0 |
| Contaminated food/drinks | 0 | 1 |
| Pets | 0 | 1 |
| Handshaking and kissing | 1 | 0 |

**Precautionary SCORE (out of 5 points):**

| **Measure** | **Yes** | **No** |
| --- | --- | --- |
| Hand washing with water | 1 | 0 |
| Hand washing with alcohol | 1 | 0 |
| Face masks | 1 | 0 |
| Avoiding crowded area | 1 | 0 |
| Avoiding handshaking | 1 | 0 |

**Treatment SCORE (out of 7 points):**

| **Measure** | **Yes** | **No** |
| --- | --- | --- |
| Currently no targeted therapy | 1 | 0 |
| Antibiotics | 0 | 1 |
| Antipyretics | 1 | 0 |
| Panadol and paracetamol | 1 | 0 |
| Drinking hot liquid | 0 | 1 |
| Herbal remedy | 0 | 1 |
| Vitamins | 0 | 1 |

**Total knowledge SCORE (out of 27 points):**

Total knowledge SCORE = (Symptoms SCORE + Transmission SCORE + Precautionary SCORE + Treatment SCORE)

**Total knowledge SCORE categories:**

- Inadequate: ≤ 18
- Average: 19-20
- Adequate: ≥ 21
